# Chronic Wasting Disease and Potential Transmission to Humans

**DOI:** 10.3201/eid1006.031082

**Published:** 2004-06

**Authors:** Ermias D. Belay, Ryan A. Maddox, Elizabeth S. Williams, Michael W. Miller, Pierluigi Gambetti, Lawrence B. Schonberger

**Affiliations:** * Centers for Disease Control and Prevention, Atlanta, Georgia, USA;; † University of Wyoming, Laramie, Wyoming, USA;; ‡ Colorado Division of Wildlife, Fort Collins, Colorado, USA;; § Case Western Reserve University, Cleveland, Ohio, USA

**Keywords:** Chronic wasting disease, prion diseases, transmissible spongiform encephalopathy, interspecies transmission, prions

## Abstract

Chronic wasting disease (CWD) of deer and elk is endemic in a tri-corner area of Colorado, Wyoming, and Nebraska, and new foci of CWD have been detected in other parts of the United States. Although detection in some areas may be related to increased surveillance, introduction of CWD due to translocation or natural migration of animals may account for some new foci of infection. Increasing spread of CWD has raised concerns about the potential for increasing human exposure to the CWD agent. The foodborne transmission of bovine spongiform encephalopathy to humans indicates that the species barrier may not completely protect humans from animal prion diseases. Conversion of human prion protein by CWD-associated prions has been demonstrated in an in vitro cell-free experiment, but limited investigations have not identified strong evidence for CWD transmission to humans. More epidemiologic and laboratory studies are needed to monitor the possibility of such transmissions.

Chronic wasting disease (CWD) is classified as a transmissible spongiform encephalopathy (TSE), or prion disease, along with other animal diseases, such as scrapie and bovine spongiform encephalopathy. The only known natural hosts for CWD are deer (*Odocoileus* species) and Rocky Mountain elk (*Cervus elaphus nelsoni*) (1,2). CWD and other TSEs are believed to be caused by a pathogenic effect on neurons of an abnormal isoform of a host-encoded glycoprotein, the prion protein. The pathogenic form of this protein appears to be devoid of nucleic acids and supports its own amplification in the host. TSEs in animals primarily occur by transmitting the etiologic agent within a species, either naturally or through domestic husbandry practices. In contrast, most such encephalopathies in humans occur as a sporadic disease with no identifiable source of infection or as a familial disease linked with mutations of the prion protein gene (3). A notable exception among the human TSEs is the variant form of Creutzfeldt-Jakob disease (vCJD), which is believed to have resulted from the foodborne transmission of bovine spongiform encephalopathy (BSE) to humans (4,5).

CWD was first identified as a fatal wasting syndrome of captive mule deer in the late 1960s in research facilities in Colorado and was recognized as a TSE in 1978 (6,7). Subsequently, this wasting disease was identified in mule deer in a research facility in Wyoming and in captive elk in both the Colorado and Wyoming facilities (6–8). The disease was first recognized in the wild in 1981, when it was diagnosed in a free-ranging elk in Colorado (1,9). By the mid-1990s, CWD had been diagnosed among free-ranging deer and elk in a contiguous area in northeastern Colorado and southeastern Wyoming, where subsequent surveillance studies confirmed it to be endemic (10). Epidemic modeling suggested that this wasting disease might have been present among free-ranging animals in some portions of the disease-endemic area several decades before it was initially recognized (10). On the basis of hunter-harvested animal surveillance, the overall prevalence of the disease in this area from 1996 through 1999 was estimated at approximately 5% in mule deer, 2% in white-tailed deer, and <1% in elk (10). In 2000, surveillance data indicated that the disease-endemic focus extended eastward into adjacent areas of Nebraska (1,11), and ongoing surveillance continues to redefine the limits of this focus.

Clinical manifestations of CWD include weight loss over weeks or months, behavioral changes, excessive salivation, difficulty swallowing, polydipsia, and polyuria (1,6–8). In some animals, ataxia and head tremors may occur. Most animals with the disease die within several months of illness onset, sometimes from aspiration pneumonia. In rare cases, illness may last for ≥1 year. In captive cervids, most cases occur in animals 2–7 years of age; however, the disease has been reported in cervids as young as 17 months and as old as >15 years of age (1). This disease can be highly transmissible within captive deer and elk populations. A prevalence of >90% was reported among mule deer in facilities where the disease has been endemic for >2 years (2,6,7,12). The mode of transmission among deer and elk is not fully understood; however, evidence supports lateral transmission through direct animal-to-animal contact or as a result of indirect exposure to the causative agent in the environment, including contaminated feed and water sources (12).

The geographic extent of CWD has changed dramatically since 1996 (2). Two largely independent and simultaneous epidemics, one in free-ranging deer and elk and another in the captive elk and deer industry, appear to represent the main framework for explaining the disease's current distribution (2). More extensive and coordinated surveillance has provided a clearer picture of its distribution over the last few years. Since 2000, the disease in free-ranging cervids has been increasingly identified outside of the original CWD-endemic areas of Colorado and Wyoming (Figure). The observed distribution seems to be related in part to natural movement of deer and elk and to commercial movement of infected animals to areas far from the disease-endemic zone. Considerable attention has been given to recent increases in the geographic spread of the disease, which in some areas is likely a result of increased surveillance rather than evidence of explosive geographic spread.

**Figure:**
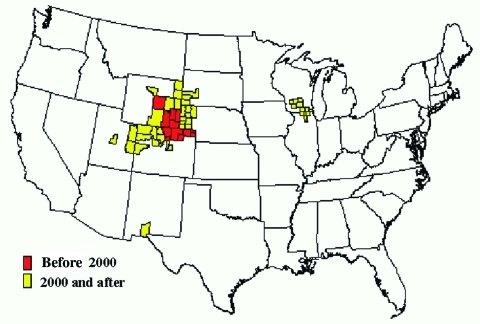
Chronic wasting disease among free-ranging deer and elk by county, United States.

No single original event or source links all wasting disease foci documented to date. Given the disease's insidious nature and the apparent duration (at least several decades) of epidemics among captive and free-ranging cervids, gaps in knowledge about its spread and distribution are not surprising, particularly within the captive deer and elk industry. However, our current knowledge cannot explain some of the distinct foci of CWD among free-ranging animals (e.g., in New Mexico and Utah). Thus, unidentified risk factors may be contributing to the occurrence of CWD among free-ranging and captive cervid populations in some areas.

## Chronic Wasting Disease in Free-ranging Deer and Elk

In 2000, surveillance of hunter-harvested deer first detected the occurrence of CWD in counties in southwestern Nebraska, adjacent to the previously recognized areas of Colorado and Wyoming that are endemic for this disease (Figure) (1,11). It was reported subsequently in other Nebraska counties, including among deer and elk in a commercial, large enclosure surrounded by a fence in northwestern Nebraska, where the prevalence of CWD was >50% (11). Free-ranging deer from areas surrounding the enclosure also tested positive for the disease but at substantially lower rates. In 2001, CWD in a free-ranging deer was identified in the southwestern part of South Dakota along the Nebraska border close to an area where the disease had been reported among captive elk (13). Since then, additional CWD-positive free-ranging deer and elk have been identified in southwestern South Dakota.

CWD in free-ranging cervids was first reported east of the Mississippi River in Wisconsin among white-tailed deer harvested in the 2001 hunting season (14). Subsequent surveillance indicated that this CWD epidemic focus was limited to several counties in the south-central region of Wisconsin, although a second focus spanning the Illinois border was also detected (15). The absence of evidence for a widespread occurrence of CWD and its low prevalence, despite a highly dense deer population, indicate that the disease probably was recently introduced into Wisconsin. Because the distance from the CWD-endemic area of Colorado-Wyoming effectively precludes eastward migration of animals as a logical source of infection, CWD in Wisconsin was more likely introduced by an imported infected cervid or some other unidentified source (14). The proximity of the Wisconsin-Illinois focus to a white-tailed deer farm with infected animals appears to support this explanation, as highlighted by the report of CWD in a previously captive white-tailed deer approximately 7 months after it escaped into the wild in southern Wisconsin (14). The disease among the captive deer herd from which the white-tailed deer escaped was demonstrated earlier, when a still-captive deer tested positive for the disease. The captive source herd was held in a facility 30–50 km from the Illinois location where CWD was recently identified in a free-ranging deer (16). In 2002, the Wisconsin Department of Natural Resources launched an ambitious culling program by providing special hunting permits to eliminate the disease in a designated "eradication zone" around the areas where it was detected (15,17). Whether such aggressive management will succeed in eliminating free-ranging foci of CWD remains to be determined.

In Colorado, the Continental Divide initially appeared to have prevented natural expansion of CWD into the western part of the state. However, in 2002, the disease was confirmed for the first time in several free-ranging deer harvested in western Colorado in an area surrounding a commercial enclosure, where entrapped mule deer tested positive for CWD. Aggressive culling of deer and elk surrounding the enclosure was initiated to prevent further spread of the disease in the western slope of Colorado. Through the 2002 hunting season, CWD-positive deer and elk continued to be identified outside of the previously defined disease-endemic area, primarily in northwestern Colorado (18). This northwestern focus appears to be discontinuous from the previously identified CWD-endemic area, although surveys conducted in 2002 demonstrated that the western and southern boundaries of that area were wider than previously believed. The ultimate source of this wasting disease in northwestern Colorado remains unidentified.

In 2002, samples from an emaciated, free-ranging mule deer found in White Sands, New Mexico, tested positive for CWD (1,19). No cervids have been held in captivity close to the area where the New Mexico deer was found, and the origin of the disease in this deer remains unknown. In addition, CWD-positive, free-ranging deer have been identified in Wyoming to the west over the Continental Divide from the known CWD-endemic zone (20). In 2003, a mature buck deer harvested in the fall of 2002 in northeastern Utah tested positive for the disease (21); additional cases have since been found in central and eastern Utah (Figure). These cases provide additional evidence for the potential spread of this wasting disease in the wild.

In Canada, CWD was first detected in free-ranging cervids (two mule deer) in 2001 in Saskatchewan; a few additional deer tested positive in 2002 and 2003 (22). Saskatchewan Environment has implemented a herd-reduction program using "control permits" to prevent further spread of the disease among free-ranging cervids.

## Chronic Wasting Disease in Captive Deer and Elk

CWD was first recognized in the captive elk industry in Saskatchewan in 1996, but subsequent investigations indicated that the most likely source of Canadian cases was captive elk imported from South Dakota prior to 1989 (2,22). Since 1996, surveillance has detected infected animals on more than 25 elk farms in Colorado, Kansas, Minnesota, Montana, Nebraska, Oklahoma, South Dakota, and Alberta, Canada, and the Republic of Korea (1,14,23,24). CWD in most of these farms was identified in the past 5 years. In 2002, the disease was detected in white-tailed deer on farms in Alberta and Wisconsin (23,25). More extensive and uniform surveillance in captive white-tailed deer is needed to determine the full extent of the disease in this industry.

Captive herds with a CWD-infected cervid are often depopulated both in Canada and the United States. Carcasses of depopulated animals are incinerated or buried in accordance with local regulations. Meat from depopulated animals has not been allowed to enter the human food and animal feed supply.

## Transmission to Other Animals

Concerns have been raised about the possible transmission of the CWD agent to domestic animals, such as cattle and sheep, which may come in contact with infected deer and elk or CWD-contaminated environments. If such transmissions were to occur, they would potentially increase the extent and frequency of human exposure to the CWD agent. In addition, passage of the agent through a secondary host could alter its infectious properties, increasing its potential for becoming more pathogenic to humans. This phenomenon may have occurred with BSE when a strain of scrapie, a possible original source of the BSE outbreak, changed its pathogenic properties for humans after infecting cattle. However, the exact origin of BSE remains unknown.

Although CWD does not appear to occur naturally outside the cervid family, it has been transmitted experimentally by intracerebral injection to a number of animals, including laboratory mice, ferrets, mink, squirrel monkeys, and goats (1,26). In an experimental study, the CWD agent was transmitted to 3 of 13 intracerebrally injected cattle after an incubation period of 22 to 27 months (27). The susceptibility of cattle intracerebrally challenged with the agent of this disease was substantially less than that observed after intracerebral scrapie challenge: nine of nine cattle succumbed to scrapie challenge after intracerebral injection (28). In ongoing experimental studies, after >6 years of observation, no prion disease has developed in 11 cattle orally challenged with the CWD agent or 24 cattle living with infected deer herds (E.S. Williams and M.W. Miller, unpub. data) (1). In addition, domestic cattle, sheep, and goat residing in research facilities in close contact with infected cervids did not develop a prion disease.

Analysis by immunohistochemical studies of the tissue distribution of prions in CWD-infected cervids identified the agent in the brain, spinal cord, eyes, peripheral nerves, and lymphoreticular tissues (Table 1) (29,30). Distribution of the CWD agent outside of the brain seems to be less widespread in elk than in deer (2). Involvement of the tonsils and peripheral nerves early in the course of experimental and natural prion infection suggests the possible involvement of the lymphoreticular and peripheral nervous systems in the pathogenesis and transmission of the disease (2,12,30,31).

**Table 1:** Deer tissues tested for the CWD agent by animal bioassay or immunohistochemical studies^a^

Tissues positive for CWD agent
Brain
Pituitary gland
Spinal cord
Eyes (optic nerve, ganglion cells, retina)
Tonsils
Lymphoid tissues (e.g., gut-associated, retropharyngeal, posterior nasal septum)
Spleen
Pancreas
Peripheral nerves (e.g., brachial plexus, sciatic nerve, vagosympathetic trunk)
Tissues negative for CWD agent
Dorsal root ganglia
Parotid and mandibular salivary glands, tongue, esophagus, small intestine, colon
Thymus
Liver
Kidneys, urinary bladder, ovary, uterus, testis, epididymis, placentomes
Myocardium, Purkinje fibers, arteries, veins
Trachea, bronchi, bronchioles, aleveolar parenchyma
Bone marrow
Thyroid gland, adrenal gland
Skeletal muscle
Skin

^a^CWD, chronic wasting disease.

## Risk for Transmission to Humans

### Epidemiologic Studies

The increasing detection of CWD in a wider geographic area and the presumed foodborne transmission of BSE to humans, resulting in cases of vCJD, have raised concerns about the possible zoonotic transmission of CWD (32). In the late 1990s, such concerns were heightened by the occurrence of CJD among three patients 30 years of age who were deer hunters or ate deer and elk meat harvested by family members (Table 2). However, epidemiologic and laboratory investigations of these case-patients indicated no strong evidence for a causal link between CWD and their CJD illness (33). None of the patients were reported to have hunted deer or eaten deer meat harvested in the CWD-endemic areas of Colorado and Wyoming. Such a history in unusually young CJD patients, if present, would have supported a causal link with CWD. Moreover, the testing of brain tissues from >1,000 deer and elk harvested from areas where the patients hunted or their venison originated did not show any evidence of CWD (33). In addition, the lack of homogeneity in the clinicopathologic manifestation and codon 129 of the prion protein gene among the three patients suggested that their illnesses could not be explained by exposure to the same prion strain. In vCJD, homogeneity of the genotype at codon 129 and the clinical and pathologic phenotype were attributed to the patients' exposure to the same prion strain, the agent of BSE.

**Table 2:** Creutzfeldt-Jakob disease patients investigated for a possible causal link of their illness with chronic wasting disease of deer and elk, United States^a^

Case no.	Age at death (y)	Year of death	Codon 129	Western blot	Final diagnosis	Eating of venison from CWD-endemic area
1	25	2001	M/V	Type 1	GSS 102	Yes
2	26	2001	M/M	Type 2	CJD	No
3^b^	28	2002	nd	nd	GSS 102	No
4	28	1997	M/M	nd	CJD	No
5	28	2000	M/V	Type 1	CJD	No
6	30	1999	V/V	Type 1	CJD	No
7	54	2002	V/V	Type 2	CJD	No
8^c^	55	1999	M/M	Type 1	CJD	No
9^d^	61	2000	M/M	Type 1	CJD	Yes
10	63	2002	V/V	Type 1	CJD	No
11^e^	64	2002	M/M	Type 1	CJD	Yes
12	66	2001	M/M	Type 1	CJD	No

^a^CWD, chronic wasting disease; GSS, Gerstmann-Sträussler-Scheinker syndrome; CJD, Creutzfeldt-Jakob disease; nd, not done. ^b^Immunohistochemical analysis of postmortem brain tissue was consistent with GSS, and a GSS 102 mutation was confirmed in the family. ^c^Investigated as part of a cluster of three case-patients who participated in "wild game feasts" in a cabin owned by one of the decedents. ^d^Patient grew up in an area known to be endemic for CWD and ate venison harvested locally; however, the CJD phenotype fits the most common form of sporadic CJD. ^e^Patient may have been successful in harvesting two deer since 1996 from a CWD-endemic area, but both deer tested negative for CWD.

In 2001, the case of a 25-year-old man who reportedly died of a prion disease after an illness lasting ≈22 months was investigated (Table 2). Although this man had hunted deer only rarely, his grandfather hunted deer and elk throughout much of the 1980s and 1990s and regularly shared the venison with the case-patient's family. The grandfather primarily hunted in southeastern Wyoming, around the known CWD-endemic area. The case-patient's illness began with a seizure and progressed to fatigue, poor concentration, and depression. Memory loss, ataxia, speech abnormalities, combative behavior, and recurrent seizures also developed. Histopathologic, immunohistochemical, and Western blot testing of brain autopsy samples confirmed a prion disease diagnosis. Analysis of the prion protein gene indicated a P102L mutation coupled with valine at the polymorphic codon 129 in the mutant allele, confirming a diagnosis of Gerstmann-Sträussler-Scheinker syndrome (GSS). This case-patient was unusually young even for a person with a GSS P102L mutation. It remains unknown whether the possible exposure of the case-patient to CWD-infected venison potentially contributed to the early onset of his prion disease.

In 2001, two additional CJD patients 26 and 28 years of age were reported from a single state (Table 2) (34). The patients grew up in adjacent counties and had illness onset within several months of each other. As a result of this fact and their unusually young age, a possible environmental source of infection, including exposure to CWD-infected venison, was considered. One of the patients died after an illness lasting 5–6 months that was characterized by progressive aphasia, memory loss, social withdrawal, vision disturbances, and seizure activity leading to status epilepticus and induced coma. Histopathologic, immunohistochemical, and Western blot testing of brain biopsy and autopsy samples confirmed a CJD diagnosis. The patient's disease phenotype corresponded to the MM2 sporadic CJD subtype reported by Parchi et al. (35). This patient did not hunt, and family members provided no history of regularly eating venison. The patient may have occasionally eaten venison originating from the Upper Peninsula of Michigan while away from home during his college years. However, ongoing surveillance has not detected CWD in Michigan deer (36).

The second patient died from an illness lasting <16 months. The patient's illness began with behavioral changes, including unusual outbursts of anger and depression. Confusion, memory loss, gait disturbances, incontinence, headaches, and photophobia also developed. Western blot analysis of frozen brain biopsy tissue confirmed a prion disease diagnosis. Immunohistochemical analysis of brain tissue obtained after the patient's death showed prion deposition consistent with GSS. A prion protein gene analysis could not be performed because appropriate samples were lacking. However, prion protein gene analysis of a blood sample from one of the patient's parents indicated a GSS P102L mutation. The patient did not hunt but may have eaten venison from Michigan once when he was 1–2 years old. The GSS diagnosis greatly reduced the likelihood that the two patients reported from adjacent counties had disease with a common origin.

Recently, rare neurologic disorders resulting in the deaths of three men who participated in "wild game feasts" in a cabin owned by one of the decedents created concern about the possible relationship of their illnesses with CWD (Table 2) (37). Two of the patients reportedly died of CJD, and the third died from Pick's disease. More than 50 persons were identified as possibly participating in these feasts; the three patients were the only participants reported to have died of a degenerative neurologic disorder. Reanalysis of autopsy brain tissues from the three patients at the National Prion Disease Pathology Surveillance Center indicated that two of them had no evidence of a prion disease by immunohistochemical analysis. CJD was confirmed in the third patient, who had clinicopathologic, codon 129, and prion characteristics similar to the most common sporadic CJD subtype (MM1/MV1) (35). This patient participated in the feasts only once, perhaps in the mid-1980s. In addition, the investigation found no evidence that the deer and elk meat served during the feasts originated from the known CWD-endemic areas of Colorado and Wyoming.

In 2003, CJD in two deer and elk hunters (54 and 66 years of age) was reported (38). The report implied that the patients had striking neuropathologic similarities and that their illness may represent a new entity in the spectrum of prion diseases. A third patient (63 years of age), who was also purported to have been a big game hunter, was subsequently reported from the same area. However, none of the three patients were reported to have eaten venison from the CWD-endemic areas of the western United States. The 66-year-old patient hunted most of his life in Washington State. Although information about the 54-year-old patient was limited, there was no evidence that he hunted in CWD-endemic areas. The third patient was not a hunter but ate venison harvested from Pennsylvania and Washington. The neuropathologic changes, Western blot profile, and genotype at codon 129 of the three patients each fit the MM1, VV1, or VV2 sporadic CJD subtype, indicating absence of phenotypic similarity among the cases or atypical neuropathologic features (35).

To date, only two nonfamilial CJD cases with a positive history of exposure to venison obtained from the known CWD-endemic areas have been reported. One of the patients was a 61-year-old woman who grew up in an area where this disease is known to be endemic, and she ate venison harvested locally. She died in 2000, and analysis of autopsy brain specimens confirmed that the patient's CJD phenotype fit the MM1 subtype, with no atypical neuropathologic features. The second patient was a 66-year-old man who was reported to have eaten venison from two deer harvested in a CWD-endemic area. Both deer tested negative for CWD, and the patient's illness was consistent with the MM1 CJD phenotype.

Despite the decades-long endemicity of CWD in Colorado and Wyoming, the incidence of CJD and the age distribution of CJD case-patients in these two states are similar to those seen in other parts of the United States. From 1979 to 2000, 67 CJD cases from Colorado and 7 from Wyoming were reported to the national multiple cause-of-death database. The average annual age-adjusted CJD death rate was 1.2 per million persons in Colorado and 0.8 in Wyoming. The proportion of CJD patients who died before age 55 in Colorado (13.4%) was similar to that of the national (10.2%). The only CJD case-patient <30 years of age in Colorado had iatrogenic CJD linked to receipt of human growth hormone injections. CJD was not reported in persons <55 years of age in Wyoming during the 22-year surveillance period.

### Laboratory Studies

The possible interspecies transmission of prions can be assessed with laboratory methods. In BSE and vCJD, several laboratory studies provided crucial evidence that helped establish a causal link between the two diseases (39–41). These studies characterized the molecular similarities of the agents causing BSE and vCJD and determined the lesion profile and incubation period patterns of different panels of mice inoculated by the two agents. Limited laboratory studies have been performed to molecularly characterize CWD-associated prions and to compare them with prions from human case-patients and other species. Strain typing studies involving wild-type inbred mice indicated that the CWD agent from a mule deer produced incubation-period and brain-lesion profiles different from those produced by the agents causing BSE and scrapie (39,42). These same strain-typing techniques had identified the similarities of the etiologic agents of BSE and vCJD, providing strong laboratory evidence for a link between the two diseases.

In human prion diseases, two major types of the proteinase-K–resistant prion protein fragment have been identified on the basis of their molecular size by one-dimensional immunoblot analysis: type 1 migrating at 21 kDa and type 2 at 19 kDa (35). N-terminal protein sequencing indicated that the cleavage site of the type 1 fragment is generally at residue 82 and that of type 2 is at residue 97 (43). Prion strain diversity is believed to be encoded in the three-dimensional conformation of the protein, which determines the cleavage site and molecular size of proteinase-K–treated prion fragment, indicating that the difference in molecular size may correlate with strain differences. However, one-dimensional immunoblot analysis may not identify more subtle differences that may influence the conformation of different prion strains. Analysis of the glycoform ratios of prion fragments and application of a two-dimensional immunoblot may help further identify these subtle differences. On one-dimensional immunoblot analysis, the prion fragment from several CWD-infected deer and elk migrated to 21 kDa, corresponding to the type 1 pattern. This specific type has been identified in most cases of sporadic CJD in the United States. However, the deer and elk prion fragment differs from that in sporadic CJD cases in the glycoform ratio. In the CWD-associated prion fragment, the diglycosylated form was predominant, but in the CJD-associated prions, the monoglycosylated form was predominant. Preliminary analysis using two-dimensional immunoblot indicated that the CWD-associated prion fragment exhibited patterns different from that of the CJD-associated prion fragment from a human patient with the type 1 pattern (S. Chen, pers. comm.). Although analysis of more samples from cervids and humans is needed before meaningful conclusions can be made, these molecular techniques could potentially be used to study the similarities or differences in prion strains from cervids and humans with possible exposure to CWD.

The likelihood of successful interspecies transmission of prion diseases is influenced by the degree of homology of the infecting prion with that of the host endogenous prion protein. Such observations have given rise to the concept of a "species barrier," which would need to be overcome before an infecting prion strain caused disease in a recipient host. In vitro cell-free conversion reaction experiments have been developed to assess the degree of molecular compatibility of disease-associated prions from one species with normal prion protein obtained from a different species (44,45). Such experiments specifically assess the likelihood that an infecting prion would potentially initiate the formation and propagation of pathogenic prions if it came in contact with normal prion protein. A cell-free conversion experiment indicated that CWD-associated prions can convert human prion protein into its abnormal conformer, albeit at a very low rate (44). The efficiency of this conversion was >14-fold weaker than the homologous conversion of cervid prion protein and >5-fold weaker than the homologous conversion induced by CJD-associated prions. A similar low efficiency conversion of human prion protein by bovine- and scrapie-associated prions was also reported (44,45). Although a high level of compatibility of prions in in vitro conversion reactions is believed to correlate with in vivo transmissibility of the agents, the threshold of compatibility efficiency below which no natural transmission should be anticipated is unknown. A low level of compatibility of infecting prions and host prion protein does not necessarily rule in or out natural interspecies transmission of prion diseases. However, the comparably low-level in vitro conversion of bovine prion protein by CWD-associated prions is consistent with the relative in vivo resistance of cattle to CWD under all but the most extreme experimental challenges. In addition, several other factors may determine the in vivo transmission of disease-associated prions, including dose, strain of the agent, route of infection, stability of the agent inside and outside the host, and the efficiency of agent delivery to the nervous system (44,46).

## Conclusions

The lack of evidence of a link between CWD transmission and unusual cases of CJD, despite several epidemiologic investigations, and the absence of an increase in CJD incidence in Colorado and Wyoming suggest that the risk, if any, of transmission of CWD to humans is low. Although the in vitro studies indicating inefficient conversion of human prion protein by CWD-associated prions raise the possibility of low-level transmission of CWD to humans, no human cases of prion disease with strong evidence of a link with CWD have been identified. However, the transmission of BSE to humans and the resulting vCJD indicate that, provided sufficient exposure, the species barrier may not completely protect humans from animal prion diseases. Because CWD has occurred in a limited geographic area for decades, an adequate number of people may not have been exposed to the CWD agent to result in a clinically recognizable human disease. The level and frequency of human exposure to the CWD agent may increase with the spread of CWD in the United States. Because the number of studies seeking evidence for CWD transmission to humans is limited, more epidemiologic and laboratory studies should be conducted to monitor the possibility of such transmissions. Studies involving transgenic mice expressing human and cervid prion protein are in progress to further assess the potential for the CWD agent to cause human disease. Epidemiologic studies have also been initiated to identify human cases of prion disease among persons with an increased risk for exposure to potentially CWD-infected deer or elk meat (47). If such cases are identified, laboratory data showing similarities of the etiologic agent to that of the CWD agent would strengthen the conclusion for a causal link. Surveillance for human prion diseases, particularly in areas where CWD has been detected, remains important to effectively monitor the possible transmission of CWD to humans. Because of the long incubation period associated with prion diseases, convincing negative results from epidemiologic and experimental laboratory studies would likely require years of follow-up. In the meantime, to minimize the risk for exposure to the CWD agent, hunters should consult with their state wildlife agencies to identify areas where CWD occurs and continue to follow advice provided by public health and wildlife agencies. Hunters should avoid eating meat from deer and elk that look sick or test positive for CWD. They should wear gloves when field-dressing carcasses, bone-out the meat from the animal, and minimize handling of brain and spinal cord tissues. As a precaution, hunters should avoid eating deer and elk tissues known to harbor the CWD agent (e.g., brain, spinal cord, eyes, spleen, tonsils, lymph nodes) from areas where CWD has been identified.
